# The mouse gingiva and HIF-1α, a key gene of hypoxic environment, as tools for post-mortem time estimation

**DOI:** 10.1371/journal.pone.0311050

**Published:** 2024-11-15

**Authors:** Salomé Mascarell, Coralie Torrens, Caroline Andrique, Asmaa Foda, Tania Delabarde, Bertrand Ludes, Anne-Margaux Collignon, Anne Poliard

**Affiliations:** 1 Imaging and Biothérapies Laboratory URP2496, Université Paris Cité, Orofacial Pathologies, Dental School, Montrouge, 92120, France; 2 Dental Medicine Departments, AP-HP, Pitié-Salpêtrière and Louis Mourier Hospitals, Université Paris Cité, Paris, 75002, France; 3 Institut Médico-Légal, Paris, 75012, France; 4 Université Paris Cité-CNRS UMR 8045 Babel, Paris, 75005, France; School of Medicine, Tokyo Women’s Medical University, JAPAN

## Abstract

The post-mortem interval (PMI) is the time elapsed between the death of an individual and its forensic examination. It is a crucial information for judicial authorities, but current techniques still cannot establish a precise time interval. Novel approaches are therefore required. Recently, gingival tissue has emerged as interesting for forensic analysis thanks to the protection offered by lips to this tissue, limiting the influence of environmental factors. It is also easily accessible, and its sampling is minimally invasive even in the presence of *rigor mortis*. Moreover, the expression of HIF-1α, a master mediator of the hypoxic environment, has been described in gingival samples at different post-mortem (PM) times. We have hypothesized that the time-dependent post-mortem expression of HIF-1α could serve as a biomarker to more accurately predict the PMI. Our analyses were performed in an animal model, the mouse, where environment can be precisely controlled. Therewith, gingival tissue morphology was evaluated through histochemical staining and HIF-1α expression was analyzed by qPCR, western blots and immunofluorescence at different post-mortem times (0h to 100h). Our results showed (a) a global post-mortem stability of gingival tissue (b) a rapid increase in *HIF-1α* mRNA expression in the short post-mortem times followed by a slow decrease in transcript expression until 100h PM (c) an expression of the HIF- 1α protein and its degradation products, that follows the mRNA pattern (d) the presence of HIF-1α protein in the epithelial and connective layers of the tissue, with signal accumulation in both gingival strata until at least 32h post-mortem. This pilot study thus validated the mouse and the gingival tissue as models for post-mortem analyses, as well as for studying the fate of proteins such as HIF-1α. Transferring these approaches to human subjects may provide a more accurate estimate of PMI.

## Introduction

Post-mortem interval (PMI) is the time elapsed between the death of an individual and its discovery. Establishing the most precise time of death is an important and critical step in forensic investigation [1]. To estimate the PMI, techniques focus primarily on the predictable changes that occur in the body after death [2]. A few days after death, depending on ambient temperature, putrefaction takes place. The first sign of putrefaction is the appearance of a greenish stain on the abdomen. The action of bacteria generates gas to be released, causing tissues (mainly in the abdomen and skin) to swell. Insects are rapidly attracted by the produced gases. Analysis of insect species, stage of development and their specific role in decomposition is called forensic entomology and is a well-known science for determining PMI of decomposing body [3]. In a recent death context (less than 5 days), forensic scientists rely primarily on Vibert’s triad: body temperature, *rigor mortis*, and *livor mortis*. Globally, after death, many physico-chemical changes can occur that lead to the dissolution of soft tissue, and cellular changes are noted accordingly [4]. The main limitation of all existing techniques is their non-reproducibility due to exogenous and endogenous factors that will cause values to vary from one individual to another and from one environment to another. Therefore, it remains one of the most difficult variables to determine, and the different approaches currently used generally result in large time intervals accuracy of 3 to 5 hours [5]. Finding more sensitive strategies, would constitute an additional support to precise PMI. Among them, assessing post-mortem changes with specific biochemical markers [6] and/or combining histological, ultrastructural, enzymatic, degradomic, immunohistochemical and molecular analyses [2,7–9] could provide new elements for PMI’s estimation and limit the examiner bias existing with traditional methods [6]. Recently, forensic research has begun focusing on studying the stability of various proteins in post-mortem tissues as a means to improve PMI determination, reviewed in Sacco and al. 2022 [10]. Indeed, PM proteins undergo modifications and degradation, the kinetics of which could enhance PMI determination, when used alongside more classical parameters [10]. In this context, the choice of tissue to sample is also very important. While skeletal or cardiac muscle tissue has been the most studied for intermediate PM times, recently, the gingival tissue has emerged as another compelling and well-suited candidate in forensic analysis [11–13]. Indeed, the gingiva is easily accessible and tissue collection is minimally invasive, even in cases of *rigor mortis*. Additionally, the influence of environmental factors is significantly limited as this tissue is covered by the lips. Samples can be collected from areas distant from the attached gingiva, less affected by periodontal diseases, regardless of the individual’s oral and dental condition prior to death.

Between 2—and 4-hours post-mortem, ultrastructural morphological changes in the gingiva seem to be directly dependent on the length of the PMI [14]. They are observed both in the epithelium and connective layers of the tissue and visually appear to mostly result from a vacuolization process, occurring in the cellular and extracellular components [11,15]. These modifications must accompany and /or reflect the existence of other potentially valuable parameters in particular at the protein level, useful for estimating the time elapsed since death. Very few studies have, up to now, searched for such parameters in post-mortem tissues and in particular in the gingiva [13,15]. Interestingly, one of them has reported a differential expression of the hypoxia-inducible factor (HIF-1α), a master mediator of the hypoxic environment taking place after death, according to the PMIs [13]. HIF-1 consists of 2 subunits -α and -β, this protein is present as an inactive monomer in the cytoplasm. HIF-1α is then transported to the nucleus, where it can dimerize with its β-subunit. In normoxia, HIF-1α is hydroxylated, increasing its affinity with a ubiquitin ligase, resulting in polyubiquitination and proteasome-mediated degradation of HIF-1α. In hypoxic conditions, hydroxylation is inactivated and HIF-1α proteins are not degraded. HIF-1 can then play its role as a transcription factor for certain genes, acting as a real transcription regulator implicated in oxygen homeostasis [16]. This differential expression of the HIF-1α factor or other downstream components of hypoxic pathways might serve as clues for identifying new biomarkers that could help to elucidate the PMI. Therefore, to pursue this direction and to ascertain the interest of this pathway in the context of the PMI estimation, we aimed to confirm the histological and molecular changes accompanying PMI in the gingiva, in a more controlled setting than human post-mortem tissues. We have made use of the mouse model and collected gingival tissues at precise PMI, and maintained at a constant temperature (20°C). This enabled us to gather a series of data before expanding our analyses to human samples. Two sets of analyses were conducted in mouse gingival samples collected within the first 100 hours post-mortem: 1) assessing the stability of the mouse gingival tissue over a span of 4 days and in parallel 2) examining hypoxia pathways activation, through HIF-1α expression both at the transcript and protein levels.

## Materials and methods

### Tissue collection

**Fig 1:**
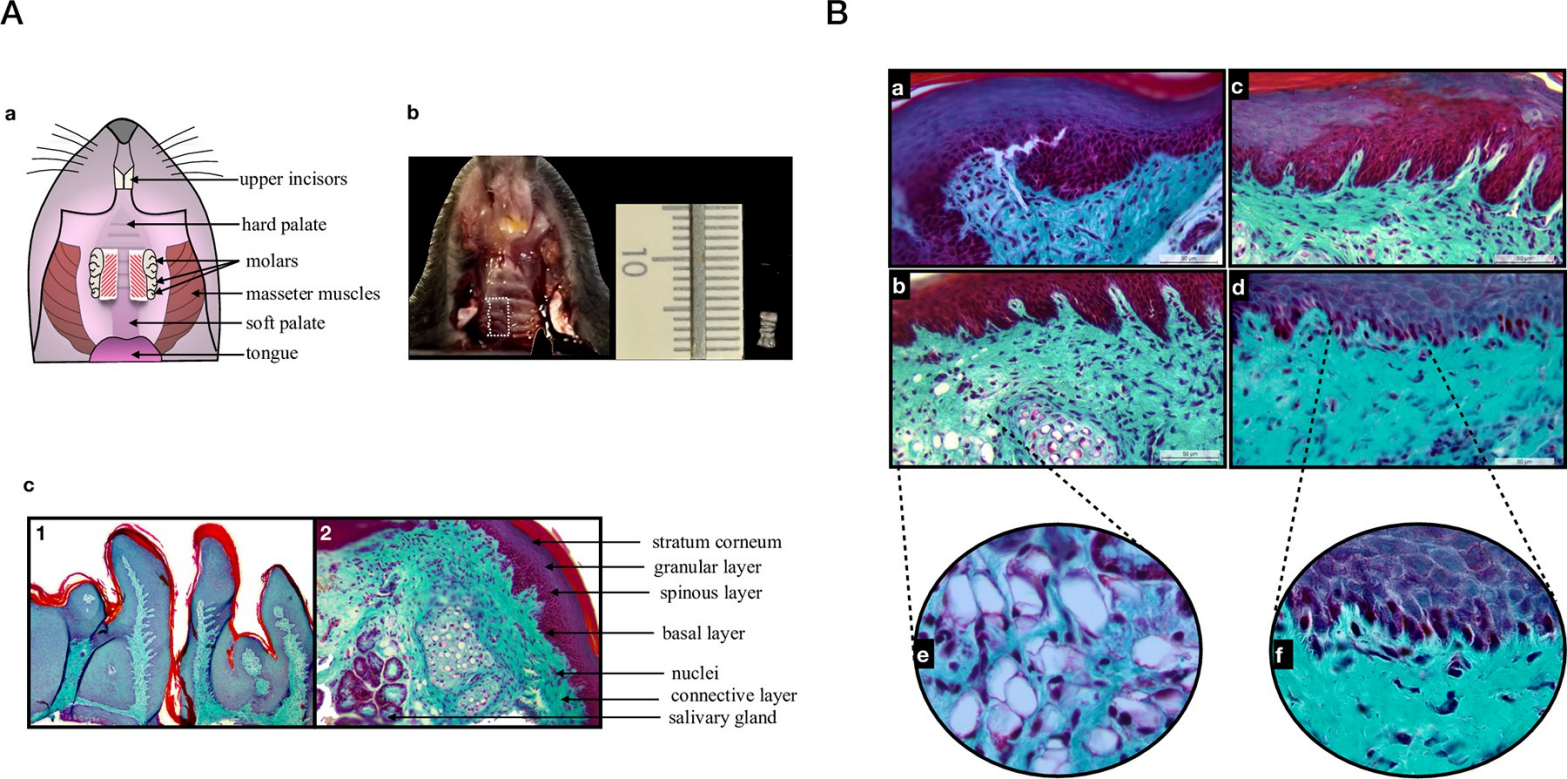
Histology of the gingival tissue. A) a) Illustration of gingival sampling areas (hatched) Source: Author. b) Occlusal view of the palatal sample zone (left) and measurement of the sample obtained (right). c) Section of gingival tissue with its main constituents at T_0_ stained with Masson’s Trichrome, (1) magnification x5: The stratified epithelium layer stained in purple and the connective layer with the collagen stained in turquoise; (2) magnification x20: Layers of the epithelium with stratum corneum, granular layer, spinous layer and basal layer, the nuclei in purple and a salivary gland visible in the connective layer. B) Masson’s Trichrome staining, magnification x40, at times a) 0, b) 1.5hours, c) 4 hours and d) 32 hours after sacrifice, showing a purple stratified epithelium with the presence of digitations in the connective tissue. Zoom on two sections showing e) the presence of vacuoles in connective tissue at 1.5h post-mortem and f) fragmented nuclei at 32h post-mortem.

All animal procedures were conducted in accordance with the ethical standards set forth by the European Communities Council Directive (animal breeding agreement C92-049-01). The protocol was approved by the Animal Experimentation Ethics committee of Université Paris Cité (Apafis agreement n°27827). For these analyses, we used 8- to 12-week-old C57BL/6J mice both female *(n = 29)* and male *(n = 28)* that were previously discarded from genomic studies due to their wild-type (WT) phenotype. All efforts were made to minimize animal pain or discomfort. After sacrifice performed by cervical elongation, gingival tissue was collected at different post-mortem times and maintained at a constant temperature (20°C). An intrasulcular incision allowed the marginal gingiva and palatal mucosa to be lifted from the tooth; the unilateral sample was removed using a stripper mesial to the first molar and distal to the third molar (Fig 1Aa and b). Immediately after sampling at a specific post-mortem time, the tissue was placed in a buffer according to the intended purpose.

For alive analysis, gingival samples from deeply anesthetized mice were used. Thirty minutes after subcutaneous injection of buprenorphine (0,1 mg/kg), anesthesia was induced by intraperitoneal injection of a mixture of Ketamine/Xylazine (respectively 80mg/kg and 10mg/kg). Deep anesthesia was controlled by testing the absence of paw reflex. Gingival samples were removed with the same protocol as for the post-mortem samples. Mice were thereafter euthanized by cervical elongation.

### Cell culture

To obtain a single-cell suspension, gingival right-side samples (*n = 3*) were digested in a solution of collagenase I 0,3%, dispase 2U/mL, penicillin-streptomycin 1%, fungizone 1% in Phosphate Buffer Saline (PBS) for 60 minutes at 37°C with agitation (150 rpm). After neutralization of the digestion solution by one volume of alpha-MEM (Gibco®) culture medium with 10% serum and 1% penicillin-streptomycin (complete medium) and a 5 minutes centrifugation, cells were resuspended in complete medium and seeded in a ventilated flask. Cells were thereafter cultured under hypoxic conditions at 5% O_2_. The gingival cells were taken at the 3 or 4^th^ passages.

### RNA extraction and Reverse Transcriptase Polymerase Chain Reaction (RT-qPCR) analysis for gene expression

Gingival right-side samples *(n = 6)* were used at each of seven post-mortem times (T_0_; T_0.5_; T_2_; T_4_; T_8_; T_32_; T_100_ hours). RNA extractions were performed with the Qiagen® RNeasy Mini Kit following the manufacturer’s instructions. Tissues were placed in RLT Plus buffer (RLT Buffer and 1% ß-mercaptoethanol, Qiagen®) and lysed using a mechanical mixer with a sterile tip (Argos® Battery-Operated Pestle Motor Mixer). Following extraction, the RNA concentration was measured using a NanoDrop (NanoVue®).

Complementary DNA synthesis -or the reverse transcriptase step of RT-PCR- was carried out following the protocol of the Verso cDNA Synthesis Kit from Thermo Fisher Scientific® after harmonization of the concentrations of the different RNA samples at 50 ng/μL.

For quantitative PCR analysis, the fluorescent probe used was a SYBR Green contained in the Master Mix qPCR compatible with the LightCycler® 96 (Roche®). A first step was performed to determine the best internal control among 3 housekeeping genes (*HGPRT*, *GAPDH* and *ACTIN*) with sample cDNA concentration ranges (25 ng/μL-20 ng/μL-15 ng/μL-10 ng/μL-5 ng/μL) and primers concentration ranges (10%-20%). Each measurement was performed in triplicate and an efficiency calculation was then performed. The housekeeping gene Actin was selected as the best stable internal control (with 94% efficiency). Relative expression analysis was performed with the LightCycler96® software and statistical analysis using GraphPad Prism9. The standard value was set at T_0_ which corresponds to the moment of sacrifice. The primers sequence were the following: *GAPDH* F: TGTGTCCGTCGTGGATCTGA / R: TTGCTGTTGAAGTCGCAGGAG; *HGPRT* F: GCTGGTGAAAAGGACCTCT / R: CACAGGACTAGAACACCTGC; *Actin* F: GTGGCATCCATGAAACTACAT / R: GGCATAGAGGTCTTTACGG; *HIF*-*1α* F: GCACTAGACAAAGTTCACCTGAGA / R: CGCTATCCACATCAAAGCAA

### Protein extraction and western blot analyses for protein expression

Gingival left-side samples *(n = 6)* were used at each of seven post-mortem times (T_0_; T_0.5_; T_2_; T_4_; T_8_; T_32_; T_100_ hours) and as alive control (*n = 3*) for western blot analysis. The tissue was dry-frozen in liquid nitrogen and mechanically ground, then lysed in RIPA buffer (0.5M Tris-HCl, pH 7.4, 1.5M NaCl, 2.5% deoxycholic acid, 10% NP-40, 10mM EDTA), supplemented with protease inhibitors (EDTA-Free 1/1000 dilution Merck® ref 539137-10VL,1X stock solution) and a cocktail of phosphatase inhibitors Sigma® (1/100 dilution ref P0044) in RIPA. It was then sonicated 3 times for 10 seconds (45 Hz). Protein concentrations were determined using the BCA Protein Assay Kit (Thermo Scientific®). Total proteins were diluted in RIPA Lysis Buffer (EMD Millipore) and Laemmeli SDS sample buffer (Thermo Scientific®) to obtain 45μg. Samples were then denatured at 95°C for 5 minutes and 45μg were deposited on the gel (Stain free gels Mini-PROTEAN TGX 4–20%, BioRad®). Electrophoresis was run at a constant voltage of 200 V-400mA until the dye front reached the bottom of the gel in TG-SDS running buffer. Proteins were transferred onto polyvinylidene fluoride (PVDF) membranes (TRANS-BLOT®, 0,45μm, BioRad®) in transfer buffer at a constant current of 90V-250 mA for 45 minutes. All blots were blocked for 45minutes in PBST (PBS 1X, 0,5% Tween, 5% Bovine Serum Albumin (BSA, Thermo scientific® ref 240401000) as a blocking agent. The following primary antibody were used: HIF-1α (monoclonal mouse- Mab1536 R&D systems), dilution 1:2000; GAPDH (polyclonal rabbit- G9545 Sigma), dilution 1:5000. All primary antibodies were diluted in 5% BSA in PBST and left on the membrane overnight at 4°C under agitation. HRP-conjugated polyclonal swine anti-rabbit was applied as secondary antibody for GAPDH (P0217, Dako®) and HRP-conjugated polyclonal goat anti-mouse for HIF (P0447, Dako®) after a series of washes. All secondary antibodies (dilution 1:10000 in 5% BSA in PBST) were applied for 1h at room temperature under agitation. Thereafter membranes were extensively washed in PBST. Antibody binding was visualized after applying the chemiluminescence substrate and photographed using a digital gel documentation system (Chemidoc BioRad®). Several exposure times were tested with ECL (Clarity**™** Western ECL Substrate BioRad®) with Chemidoc imager. Densitometry analysis was performed on three gels (S1 Fig) using the ImageJ Gel Analysis tool.

### Tissue preparation for immune- and histochemical analyses

Gingival samples *(n = 3)* were used at each of four post-mortem times (T_0_; T_1.5_; T_4_; T_32_ hours). Tissues were fixed in AntigenFix® during one hour, dehydrated in graded ethanol solutions and embedded in paraffin blocks in the transverse direction to provide a larger cutting surface. Sections were made using a microtome calibrated to 5 microns and stained using Masson’s Trichrome every ten sections.

### Immunohistochemical analyses

For immunohistochemistry, samples were deparaffinized, and nonspecific peroxidases were blocked for 10 minutes with ortho-periodic acid. Nonspecific protein bindings were blocked with a solution of 5% bovine serum albumin (BSA) during 1 h. Samples were then incubated overnight at 4°C with primary antibodies against HIF-1α (20960-1-AP -Proteintech) (dilution 1:200). The secondary antibody, a polyclonal anti-rabbit IgG/HRP Dako ® (dilution of 1:100), was incubated after a series of washes in PBST, during 1h at room temperature and washed extensively thereafter. The reaction was developed with 3.3′-diaminobenzidine tetrahydrochloride (DAB), and samples were counterstained with hematoxylin.

For immunofluorescence, a secondary polyclonal anti-rabbit antibody, Alexa-fluor 488 (Invitrogen® #A-11008) was applied in the dark for 1h (dilution 1:200) and nuclei were labeled with 1μg/ml DAPI (Invitrogen®). Negative controls were performed in parallel by omission of the primary antibodies (S3 Fig).

Mean fluorescence intensity was calculated on parallel sections covering all gingival samples, using Image J software. Measurements were made on thirty cells per layers (basal, superficial and connective layers) for each PM times.

### Statistical analysis

For the analysis of the relative expression of HIF-1α transcripts by qPCR, the ΔΔCq calculation was conducted using Actin as the reference gene and T_0_ as the control time point, utilizing Excel software. Data, including the mean, standard deviation, and number of replicates, were analyzed using GraphPad Prism9 with an ordinary one-way ANOVA test, and a significance level of p = 0.05 was applied.

For blot densitometry analysis, calculations were performed using GAPDH as the reference gene and T_0_ as the control time point. The adjusted density was determined on Excel software based on profile plot data obtained from the ImageJ Gel Analysis tool, with individual gel background subtraction for each band. Semi-quantitative values were obtained by collecting the adjusted densities from three gels for each time point and analyzing these data using an ordinary one-way ANOVA test in GraphPad Prism9, with a significance level set at p = 0.05.

For mean fluorescence intensity measurements, the circular area corresponding to each cell and their mean grey values were collected using ImageJ. Thirty cells per layers (basal and superficial layers of the epithelium and connective tissue), with comparable areas, were analyzed. The data, including the mean, standard deviation, and number of replicates, were analyzed using GraphPad Prism9 with an ordinary two-way ANOVA test, and the significance level was set at p = 0.05.

## Results

### Morphological stability of post-mortem gingival tissue

To evaluate potential alterations of the post-mortem gingival tissues, Masson’s Trichrome staining was performed on gingival tissue sections at the different post-mortem times. A T_0_ (time of death), tissue showed the characteristic structures of gingiva with a stratified epithelium divided in 4 layers with stratum corneum, granular layer, spinous layer and basal layer; epithelial stratum presents digitations into the inner layer composed by connective tissue (Fig 1Ac and 1Ba). These structures remained globally morphologically stable up to 32 hours PM with few fragmented nuclei and vacuolization zones which appeared more frequent at T_32_ PM (Fig 1Bb-c-d). Moreover, nuclei organization in post-mortem gingiva appeared similar to what has been described in a living tissue with a homogeneous distribution in connective layer and a decreasing gradient in number towards the superficial layer (Fig 1B) [17]. The observations of the relative stability of the gingival tissue structures at the various PM times led us to examine the kinetic of HIF-1α expression in this tissue at the same post-mortem times both at the transcript and protein levels.

### Post-mortem expression of HIF-1α transcripts and proteins

**Fig 2:**
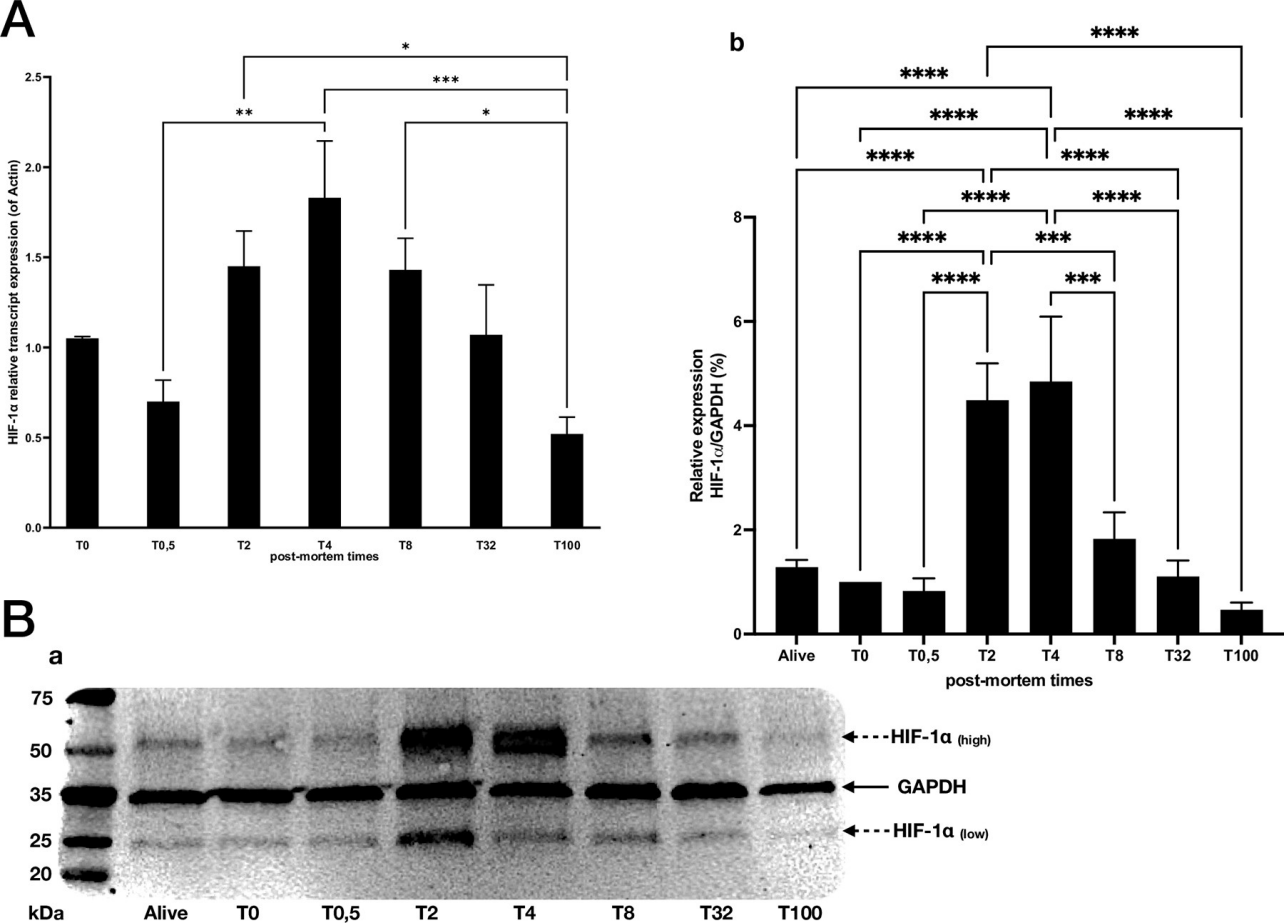
Expression of HIF-1α in qPCR and Western blot analysis at different post-mortem times. n = 6 per post-mortem time. A) HIF-1α relative transcript expression, on mouse gingival cDNA from various samples at different post-mortem times: T_0_, T_0.5_ T_2_, T_4_, T_8_, T_32_, T_100_ *p<0.05; **p<0.01; *** p<0.001. B) Western blot of HIF-1α protein expression with a) membrane photograph showing the presence of several bands corresponding to a high band of HIF-1α (50kDa), GAPDH (35kDa), and low band of HIF-1α (25kDa) at every analysis time (Alive, T_0_, T_0.5_ T_2,_ T_4_, T_8,_ T_32_ and T_100hours_), b) relative protein expression of HIF-1α, compared to GAPDH, with reference value T0: *** p<0.001; **** p<0.0001.

The expression of *HIF-1α* transcripts at the different post-mortem times was then evaluated by RT-qPCR. This expression followed a bell-shaped kinetics with a progressive increase in expression in early PM times, with a two-fold increase between T_0.5_ and T_4_, followed by a slow decrease with a two-fold decrease between T_4_ and T_32_. The expression was divided by three between T_8_ and T_100_ but still detectable at the latest PM time (Fig 2A). To assess whether the activation of *HIF-1α* mRNA expression was reflected at the protein level, a western blot analysis was performed using a specific anti HIF-1α antibody. The HIF-1α protein was detectable at all these time points with two bands at 50 and 25 kDa (Fig 2Ba). The highest band was thicker at T_2_ and T_4_ compared to the alive sample, T_0_ and T_0,5_ and progressively thinner at T_8_, T_32_ and still slightly visible at T_100_. The lowest HIF-1α bands expression followed a similar expression pattern. The 50kDa band was also visible on the positive control of HIF-1α on cells from living mouse cultured in hypoxia and an additional band at 150kDa was detected (S2 Fig). The expression of GAPDH as internal control remained essentially stable over the post-mortem times studied, with a slight decrease revealed at the later PM times. A densitometry analysis was performed on gels using the same specific anti HIF-1α antibody as previously described and GAPDH as internal control. The semi-quantitative expression evaluation of HIF-1α -to GAPDH expression- revealed a significant increase in HIF-1α at T_2_/T_4_ (four-fold factor) compared to the earliest PM times (T_0_, T_0.5_) and HIF-1α expression in the alive condition. From T_8_ onwards, the decrease was significant until it fell below the control time threshold at T_100_ (Fig 2Bb). These observations led us to determine the HIF-1α protein localization in the gingival tissue.

### Localization of the HIF-1α protein in the post-mortem gingival tissue

**Fig 3:**
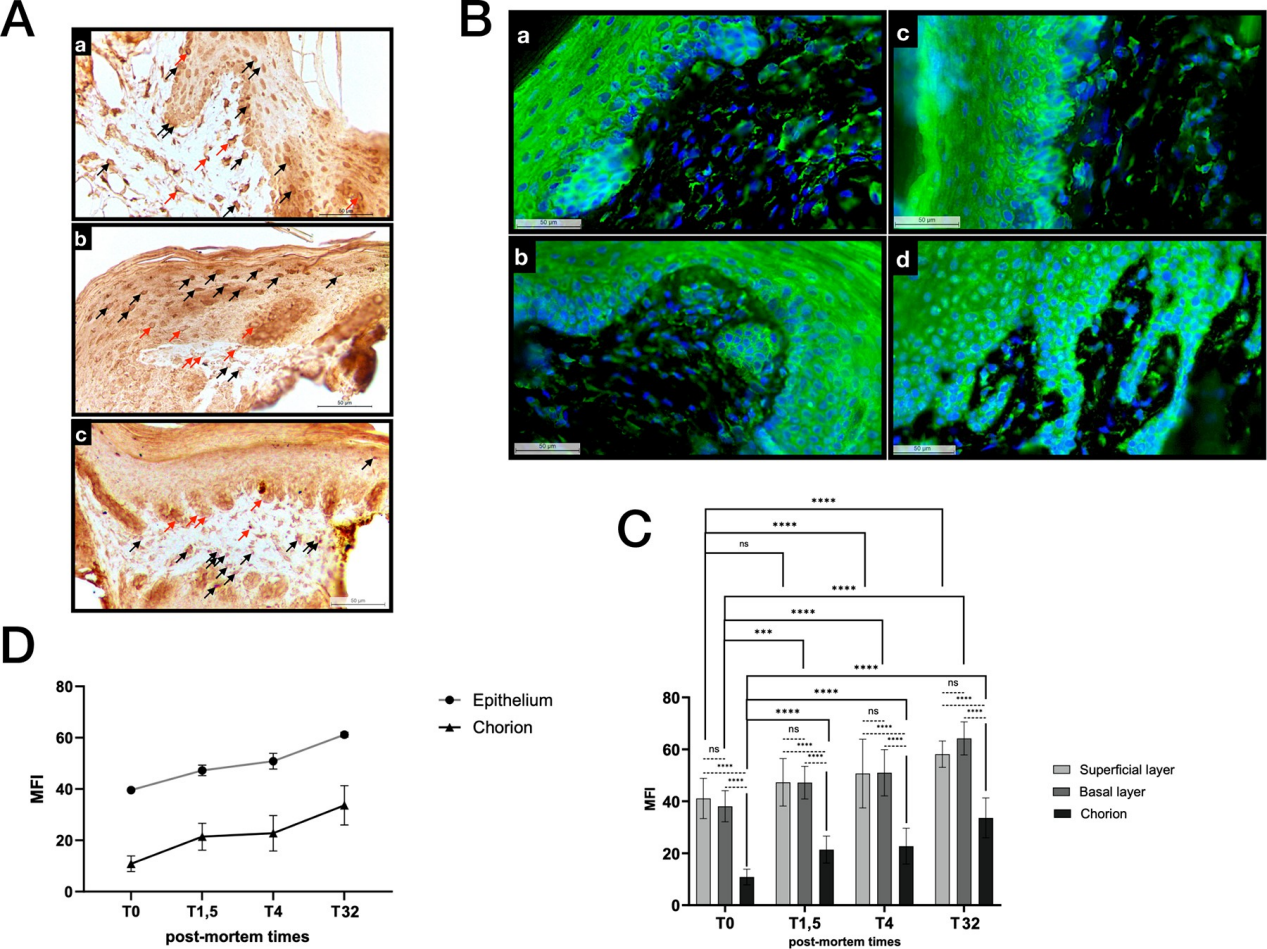
Immunohistological analysis of the post-mortem gingival tissue. A) Immunohistochemistry of HIF-1α protein at different post-mortem times a) 0, b) 1.5 hours, c) 4 hours, magnification: x40. Nuclear labeling of the protein is observed (black arrows). Presence of a polarized peri-nuclear labeling (red arrows) on one side of the nucleus only. B) Immunofluorescence of HIF-1α protein with Alexa-fluor 488 labelling and DAPI labeling for nuclei, magnification x40, at times a) 0, b) 1.5 hours, c) 4 hours and d) 32 hours after sacrifice. C-D) Semi-quantitative analysis of immunofluorescence sections. n = 3 per post-mortem time. C) Mean fluorescence intensity (MFI) at the 4 analysis times splitting the sections in three layers: Superficial, basal and connective using 2-factor tukey anova test, ***p<0.001; **** p<0.0001. D) Curves showing the evolution of HIF-1α MFI as post-mortem time progresses.

To determine the HIF-1α protein localization in the gingival tissue, an immunohistochemical analysis was first performed. It revealed a signal, in both the epithelial and connective cell layers, at all PM times. However, while all cells presented a somewhat diffuse positive signal in the epithelial layer, the labeling was heterogeneous in the connective portion of the tissue, with cells strongly labeled and others, clearly negative. In addition, in this zone, the signal was essentially nuclear or peri-nuclear (Figs 3A and S3). In order to obtain a semi-quantitative evaluation of this signal, an immunofluorescence analysis was performed. In agreement with the histochemical observations, HIF-1α labeling was observed in both gingival tissue layers up to 32h PM. The labeling appeared, as observed previously, diffuse in the epithelium and somewhat punctiform in the connective layer where HIF-1α positive and negative cells co-existed. The peri-nuclear signal localization, already revealed in the immunohistochemical analysis, concerned the majority of the gingival cells (Figs 3B and S3). A semi-quantitative analysis of the fluorescence intensity showed that the mean fluorescence intensity (MFI) was, at all times statistically greater in the superficial and basal layers of the epithelium than the signal in the connective layer. For each PM time, differences between the MFI within the two epithelial layers (basal and superficial) were not significant. Between T_0_ and T_32_ PM, a significant increase in the HIF-1α fluorescence was recorded with a two-fold increase in signal in the connective layer between T_0_ and T_1.5_. This increase concerned both the epithelial and connective layers (Fig 3C). These observations were confirmed by the MFI evolution curves. A sharp upward slope was observed between T_0_ and T_1.5_, succeeded by a more gradual slope extending from T_1.5_ to T_32_. In addition, the epithelial and connective strata appeared to evolve in parallel (Fig 3D).

## Discussion

Recently, gingiva has emerged as an interesting tissue to study in the forensics context [11–14] and the major actor of a hypoxic environment, HIF-1α, presented as a potential marker of interest for PMI determination [13]. To validate this hypothesis, we have conducted an analysis of the gingival tissue stability at different PMI and simultaneously assessed the expression of HIF-1α at both the RNA and protein level in a mouse model. Indeed, analyses of human tissues are known to be challenging due to the high environmental and individual variabilities. The utilization of animal models allows for a strict control over genetic and environmental factors thereby facilitating hypothesis evaluation. This study was thus aimed at 1) validating the behavior of the post-mortem mouse tissue as compared to its human counterpart [11,12,14] and 2) performing quantitative studies of HIF-1α expression in this defined context of the mouse model to determine whether HIF-1α could serve as a biologic marker to be correlated with PMI [13]. Studies conducted on humans and porcine cadavers have highlighted the significant role of body mass in influencing the rate of decomposition [18,19]. Given that mice are considerably lighter than humans (30g versus 60kg), this difference in weight could account for the more rapid decomposition processes observed in mice, making this animal model more compatible with laboratory analysis timeframes. In our study, gingival tissue from mice was examined up to 100 hours post-mortem (4 days), which corresponds to a putrefaction stage previously identified in small animals, characterized by hair loss, black discoloration of ruptured skin (notably on the tongue), and a strong ammonia odor [20,21]. These signs of putrefaction align with stage 3 decomposition in humans, as described by Cockle, which typically occurs at an average post-mortem interval (PMI) of 11 days [22]. Therefore, the timeframe used for analysis in mice appears relevant and applicable to human decomposition. The mouse vestibular gingiva, equivalent to the human gingiva, is very thin and highly variable in thickness therefore, gingival samples had to be collected on the palatal side. Nevertheless, the presence of a stratified epithelium and connective tissue, characteristic of the human gingiva were clearly visible in the histological analysis of the mouse gingival samples [23]. Our systematic observations of gingival sections up to 32 hours PM did not reveal any massive difference in the tissue morphology. Most cell nuclei appeared macroscopically intact even at the latest PM times of the analysis, although some vacuolization and fragmented nuclei were observed in both epithelial and connective layers. These alterations were however observed at all PM times, as also reported in the human tissue [14]. Relevantly, no major visual tissue degradation was observed up to T_32_ hours when the first clinical signs of putrefaction, such as softness of the mouse’s abdomen, started to be observed. Therefore, the mouse gingival tissue proves to be suitable for analyzing the stability and/or degradation of proteins during short term PMI.

On a molecular point of view, our data on mouse gingival tissue support the interest of the HIF-1α pathway in the context of PM time estimation as proposed for the human tissue [13]. Indeed, activation of HIF-1α expression was observed both at the transcript and protein level during the first hours following death. At the mRNA level, a *HIF-1α* rapid expression activation was recorded in the short PMIs (T_0.5_ to T_4_), in line with the results reported by Fais and al for the human tissue [13]. Thereafter, from T_4_ to T_100_ expression slowly decreased. A similar pattern of expression for the HIF-1α protein was observed in Western blot analysis. The initial expression activation is coherent with the literature showing that *HIF-1α* mRNA stability was typically increased as part of the cellular response to hypoxia. This stabilization then allowed for the sustained expression of HIF-1α protein [13]. The decrease observed after T_4_ likely reflected a progressive inactivation of the cellular machinery after death. The HIF-1α protein was detected by western blots at all time of the kinetics. However, the expected molecular weight of 150kDa [24] was only obtained in one of our controls, gingival cells cultured under hypoxic environment. At all the PM time points, two bands were present at 50 and 25 kDa. As HIF-1α is known as a rapidly degraded protein, these bands likely corresponded to stable degradation products as observed by other authors [25,26]. These bands did not appear specifically associated with the tissue post-mortem status since the same band pattern was observed in living gingiva, but rather on the protein extraction process. Surprisingly, our Western blot analyses also revealed a relative stability of GAPDH, a ubiquitous enzyme -used here as housekeeping function- up to 100h PM. This stability -up to 100h PM- was also recorded for actin, a structural protein often used as a control in living tissue (not shown). These observations thus claimed for a general good stability of the proteins in the post-mortem gingival tissue. This observed PM protein relative stability is consistent with findings in other cadaveric tissues such as human brain, skeletal muscle or porcine dental pulp [2,9,27,28]. However, authors reported that sampling these tissues was complex and not reproducible due to difficulties in accessing the tissue. In contrast, here gingival tissue sampling has proven straightforward and reproducible.

At the cellular level, HIF-1α localization and expression were primarily observed in the epithelium, with more precise and specific labeling in the connective layer. The mechanisms underlining this difference are not cleat at present. Further analyses involving specific dissection of each gingival layer should help determining whether post-mortem-dependent events specific to each layer could account for the observed differences in staining patterns. Our semi-quantitative analysis of MFI revealed an accumulation of the HIF-1α signal over the post-mortem time course, with a particularly swift increase at short time scales in agreement with the transcript and western blots analyses. Response to hypoxic stress in a living organism, is classically characterized by activation of the transcription factor HIF-1α expression and its transport of HIF-1α to the cell nuclei where it will activate a series of downstream genes [24]. In the PM mouse gingival tissue, the nuclear localization was visible in some cells but the signal was more predominantly observed around the nuclei. Such a perinuclear accumulation is compatible with HIF-1α localization in the proteasome, the cellular compartment where the protein is classically degraded in physiological conditions [29]. This pattern may reflect the fact that HIF-1α is rapidly produced in response to the hypoxia taking place upon death but that, thereafter, the cellular machinery responsible for transporting the protein within the nuclei becomes rapidly inefficient.

All together, these data on post-mortem mouse gingival tissue: 1) establish the mouse gingiva as a valuable tool to study how the environment impacts the post-mortem tissue as a first approach substitute for the human tissue and 2) ascertain the potential interest in forensics analysis of the gingival expression of HIF-1α and its downstream targets, as putative markers for PMI’s estimation. More precisely, these results support the interest of studying the kinetics of HIF-1α protein expression/degradation in relation to post-mortem times. In this context our preliminary results on human cadaveric gingival tissue confirm the observations made in the mouse model. Indeed, not only did the post-mortem gingiva displayed a morphological stability of its characteristic elements together with an integrity of the cell nuclei but also the presence of our protein of interest was revealed in both the epithelium and the connective layers up to 100h PM.

Despite these encouraging data, we acknowledge that our study, conducted on a mouse model in controlled settings, does not fully replicate typical forensic scenarios. The high variability present in human populations—such as differences in age, genetics, health status, oral hygiene and other factors—along with environmental variations like temperature, humidity, can significantly influence post-mortem parameters. Additionally, the manner of death, such as "sudden death" modeled in our mouse study by cervical elongation euthanasia, as opposed to "prolonged agony," is another important factor likely affecting PM parameters. However, our preliminary data on human samples indicate that age and oral health status do not have a significant impact on gingival protein stability. Furthermore, individual variations could certainly be restricted by working with well-defined human subgroups. Additionally, hypoxia is inherently associated with the event of death. Therefore, the activation of hypoxia signaling pathways is a universal post-mortem phenomenon, regardless of the circumstances of death, which is accounted for in our study design. Altogether our data demonstrate the interest of studying the HIF-1α pathway proteins in post-mortem gingiva as a putative new parameter for improving PMI determination.

## Conclusions

The development of new protocols for contributing to a determination of more precise and reproducible PMI is of undoubted interest to forensic scientific teams, for whom establishing the PMI remains a daily challenge during autopsies. This work constitutes a pilot study aimed at developing a novel approach to correlate the presence of specific proteins or their degradation products in gingival tissue, with the PMI. We have here demonstrated the potential of the mouse model for investigating proteins associated with the HIF-1α signaling pathway as putative biological markers for estimating PMI. Indeed, this tissue has proven to visually maintain his structure for up to 100h post-mortem. Significant variations in HIF-1α level of expression have been observed, at both the transcript and protein levels, occurring within minutes to a few hours PM, the exact time frame targeted by forensic analysis. In addition, gingival proteins displayed a good stability during the first days following death. These observations open new avenues for analyzing the HIF-1α proteasome/degradome in PM gingiva to identify proteins, or their degradation products associated with specific PMI. Ultimately, incorporating these new molecular parameters, alongside existing ones, should help refine the estimation of PMI.

## Supporting information

S1 Fig Raw images of blots gel. Gels used for densitometry analyses and as control.
(PDF)

S2 Fig Western blot analysis of HIF-1α’s expression on hypoxic cells. Western blot of HIF-1α protein expression: Membrane photograph of proteins from gingival cells isolated from living mice and cultivated under hypoxic conditions (5% O_2_), two bands corresponding to a high band of HIF-1α (150kDa) and low band of HIF-1α (50kDa).
(TIF)

S3 Fig Negative control of immunohistological analysis using post-mortem gingival tissue without labelling with the primary anti-HIF-1α’s antibody. a) Negative control of immunohistochemistry with second antibody only and b) Negative control of immunofluorescence with second antibody only and DAPI labeling for nuclei.
(TIF)

S1 Table Minimal data set. Tables of all the data used for statistical analysis in qPCR, densitometry and mean fluorescence intensity.
(XLSX)
