# Supplementary material for: The mouse gingiva and HIF-1α, a key gene of hypoxic environment, as tools for post-mortem time estimation
Source: PLoS One. 2024 Nov 15;19(11):e0311050. doi: 10.1371/journal.pone.0311050 (PMC11567532; doi:10.1371/journal.pone.0311050)
Supplement: S1 Fig — Gels used for densitometry analyses and as control. (PDF) [file pone.0311050.s001.pdf]

GEL 1

Photographed using a digital gel documentation system (Chemidoc BioRad®) using the Chemidoc imager

Exposure time = 70 seconds

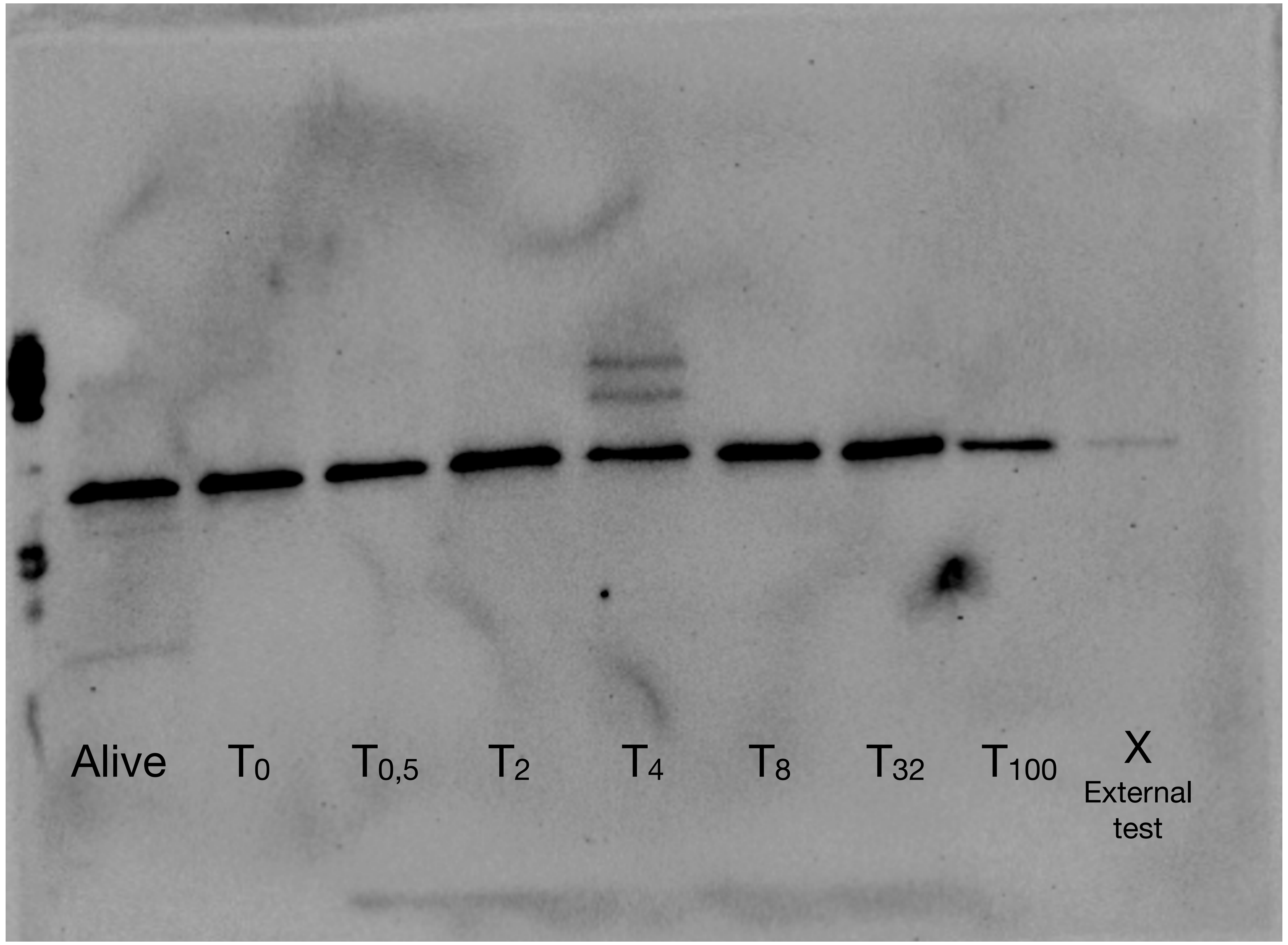

GAPDH polyclonal rabbit- G9545 Sigma

Exposure time = 200 seconds

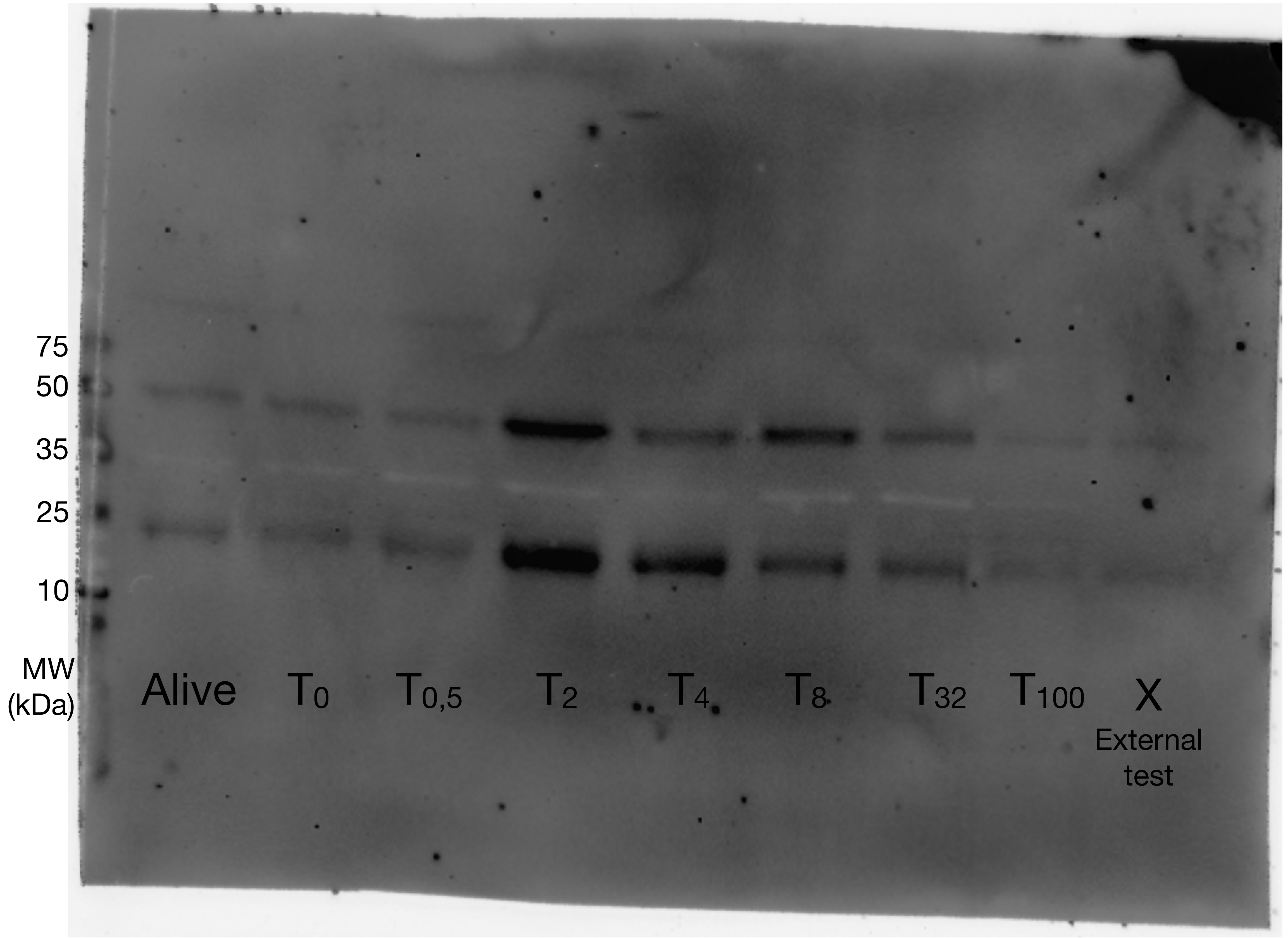

HIF monoclonal mouse- Mab1536 R&D systems

GEL 2

Photographed using a digital gel documentation system (Chemidoc BioRad®) using the Chemidoc imager

Exposure time = 4.3 seconds

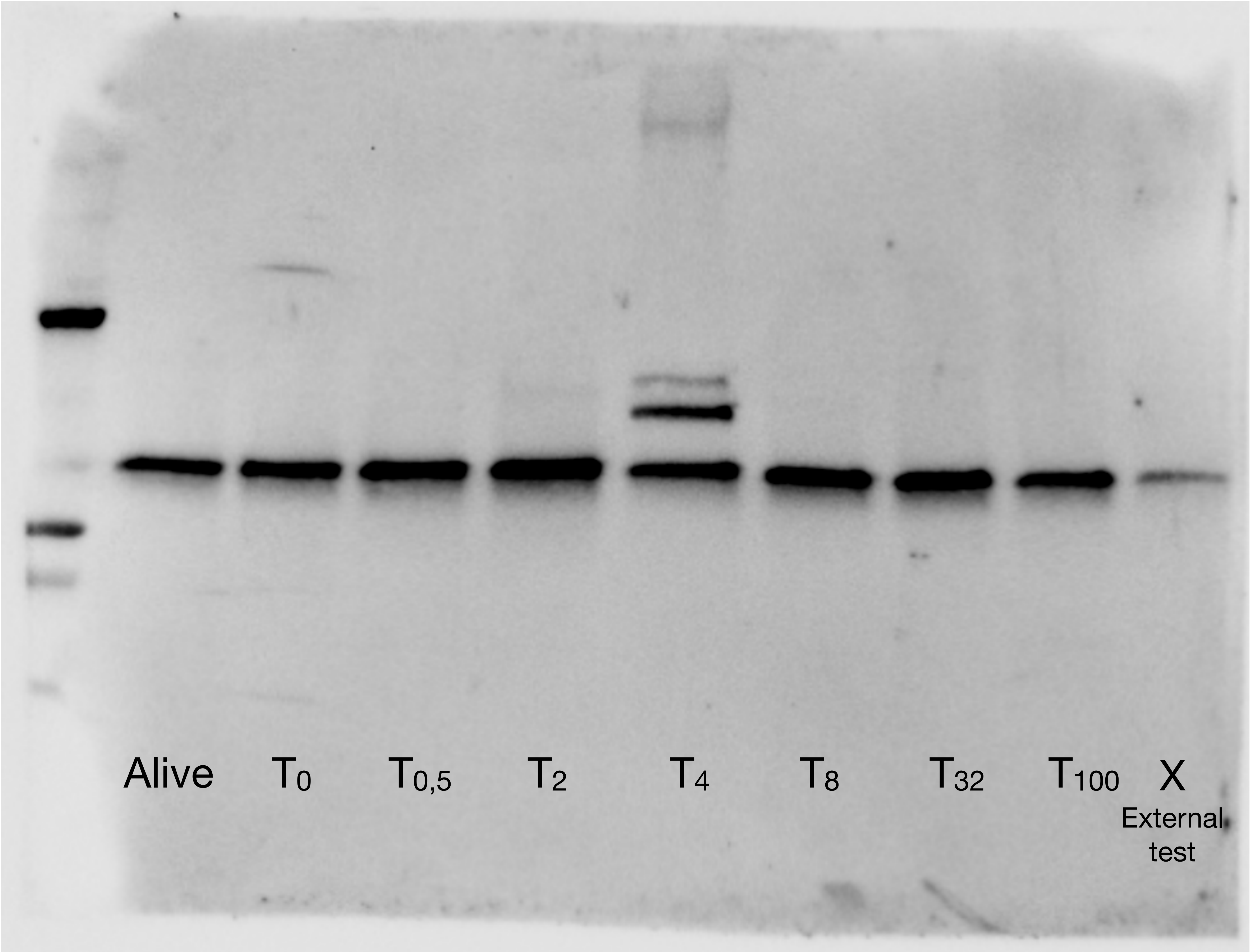

GAPDH polyclonal rabbit- G9545 Sigma

Exposure time = 30 seconds

75
50
35
25
10
MW (kDa)

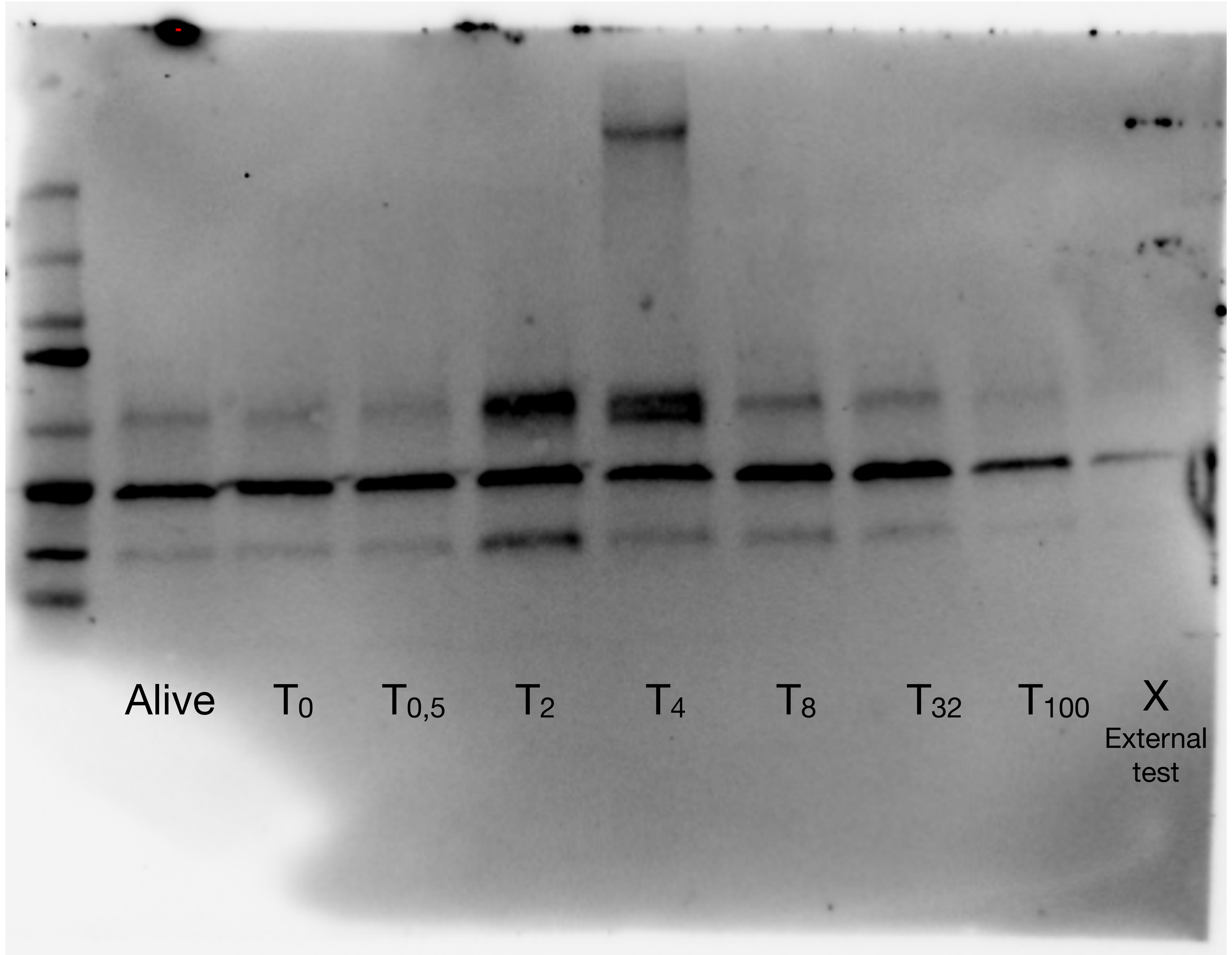

HIF monoclonal mouse- Mab1536 R&D systems with remaining GAPDH at 35kDa

GEL 2

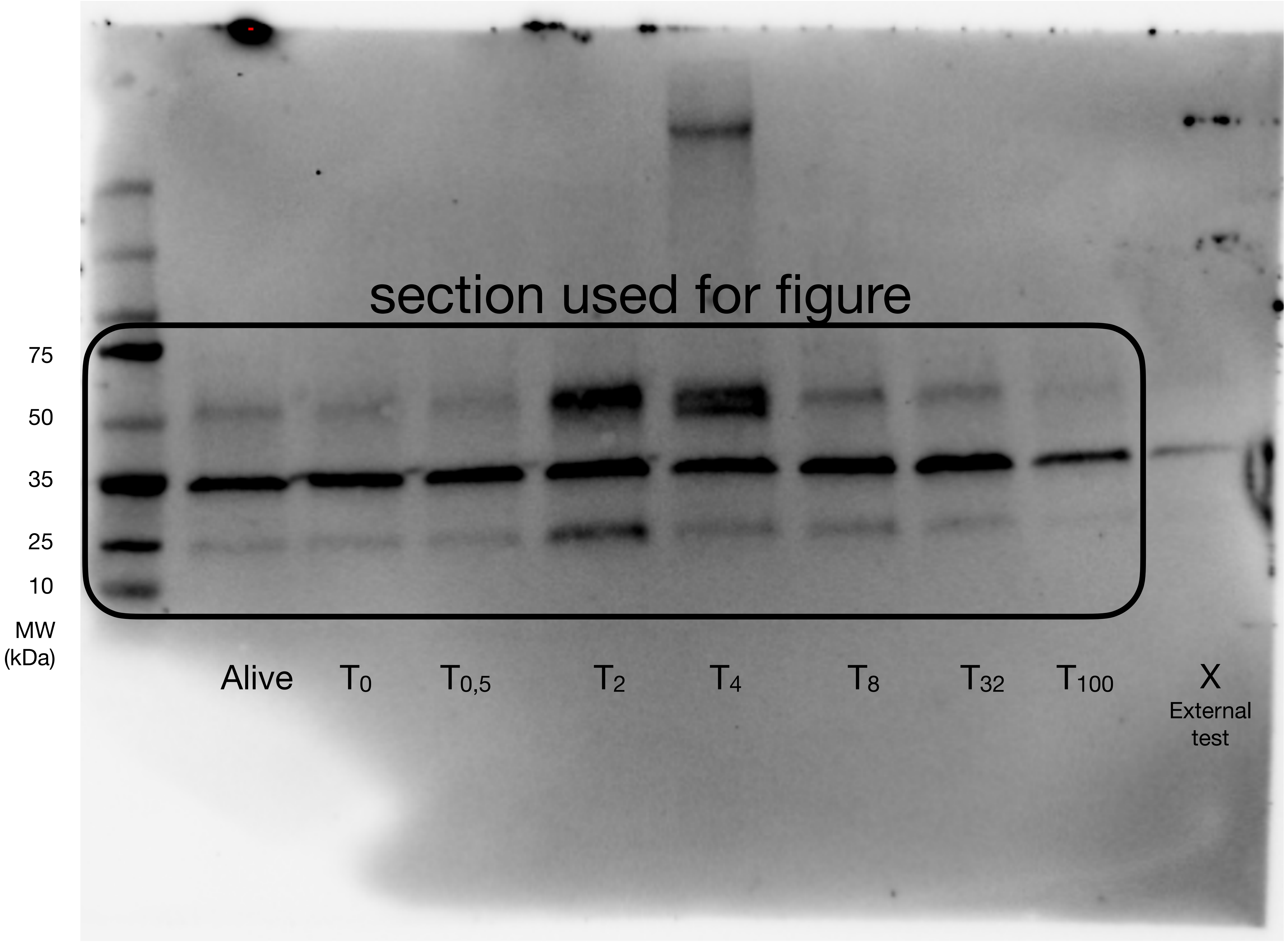

HIF monoclonal mouse- Mab1536 R&D systems with
remaining GAPDH at 35kDa

GEL 3

Photographed using a digital gel documentation system (Chemidoc BioRad®) using the Chemidoc imager

Exposure time = 186 seconds

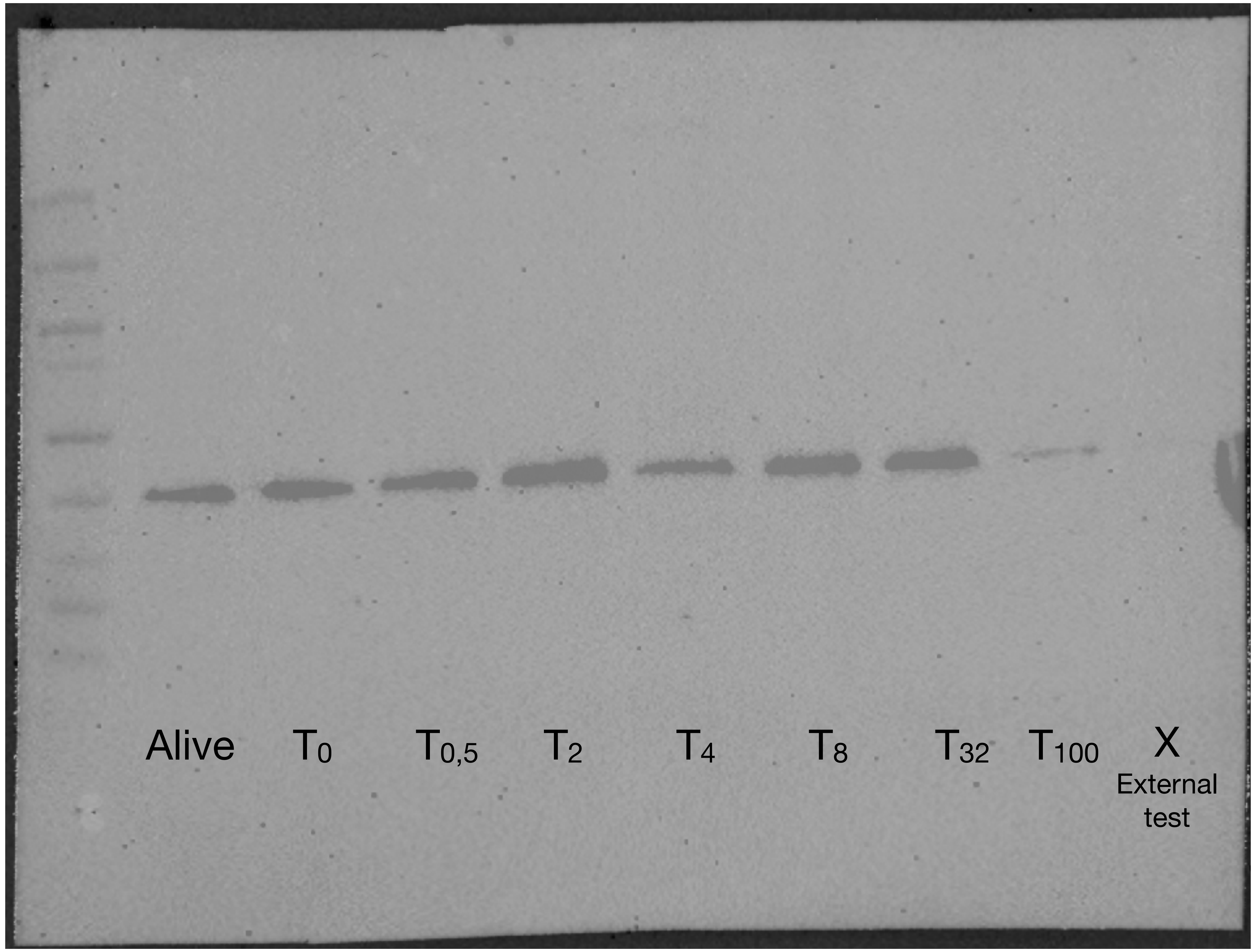

GAPDH polyclonal rabbit- G9545 Sigma

Exposure time = 120 seconds

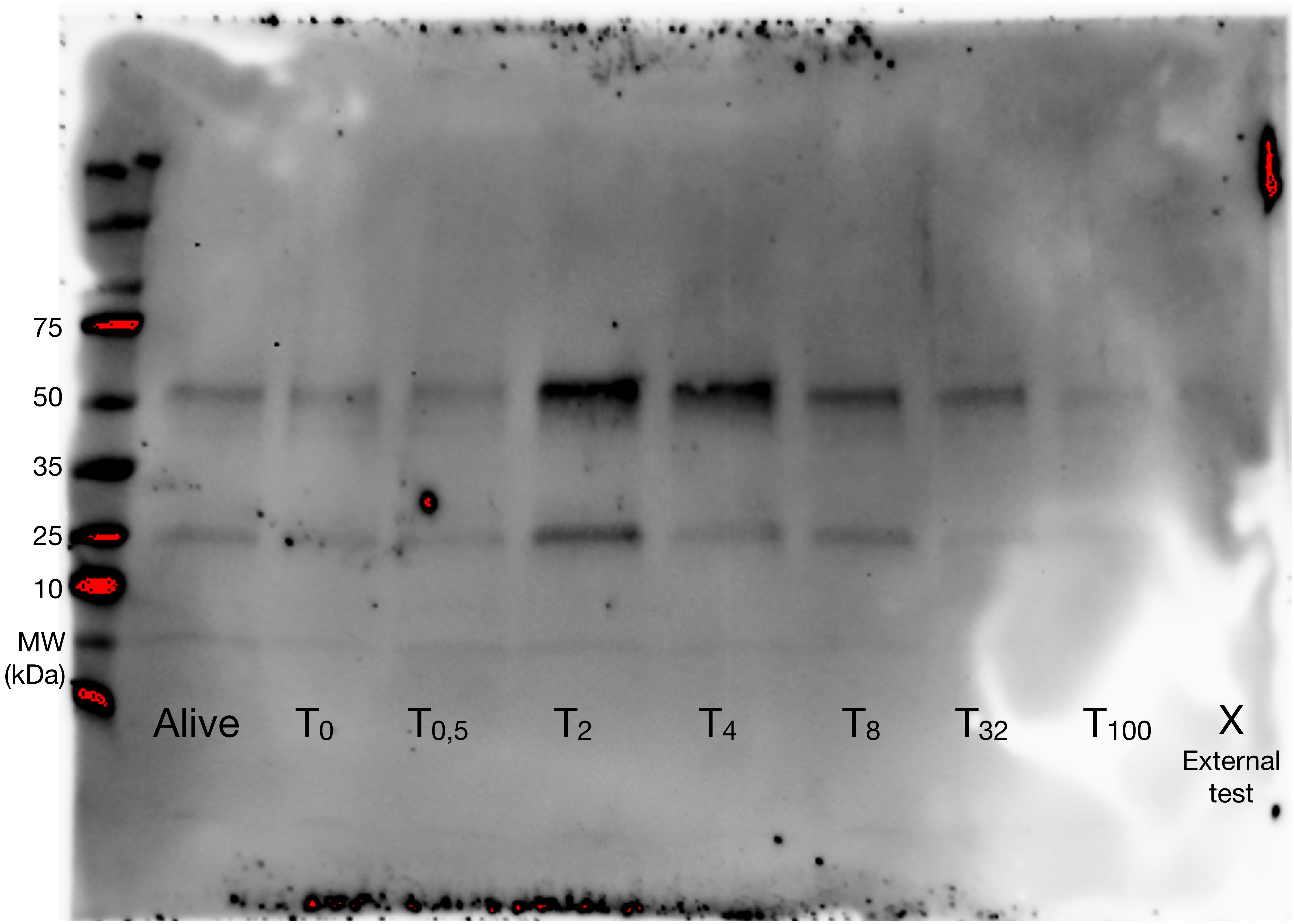

HIF monoclonal mouse- Mab1536 R&D systems

Exposure time = 200 seconds

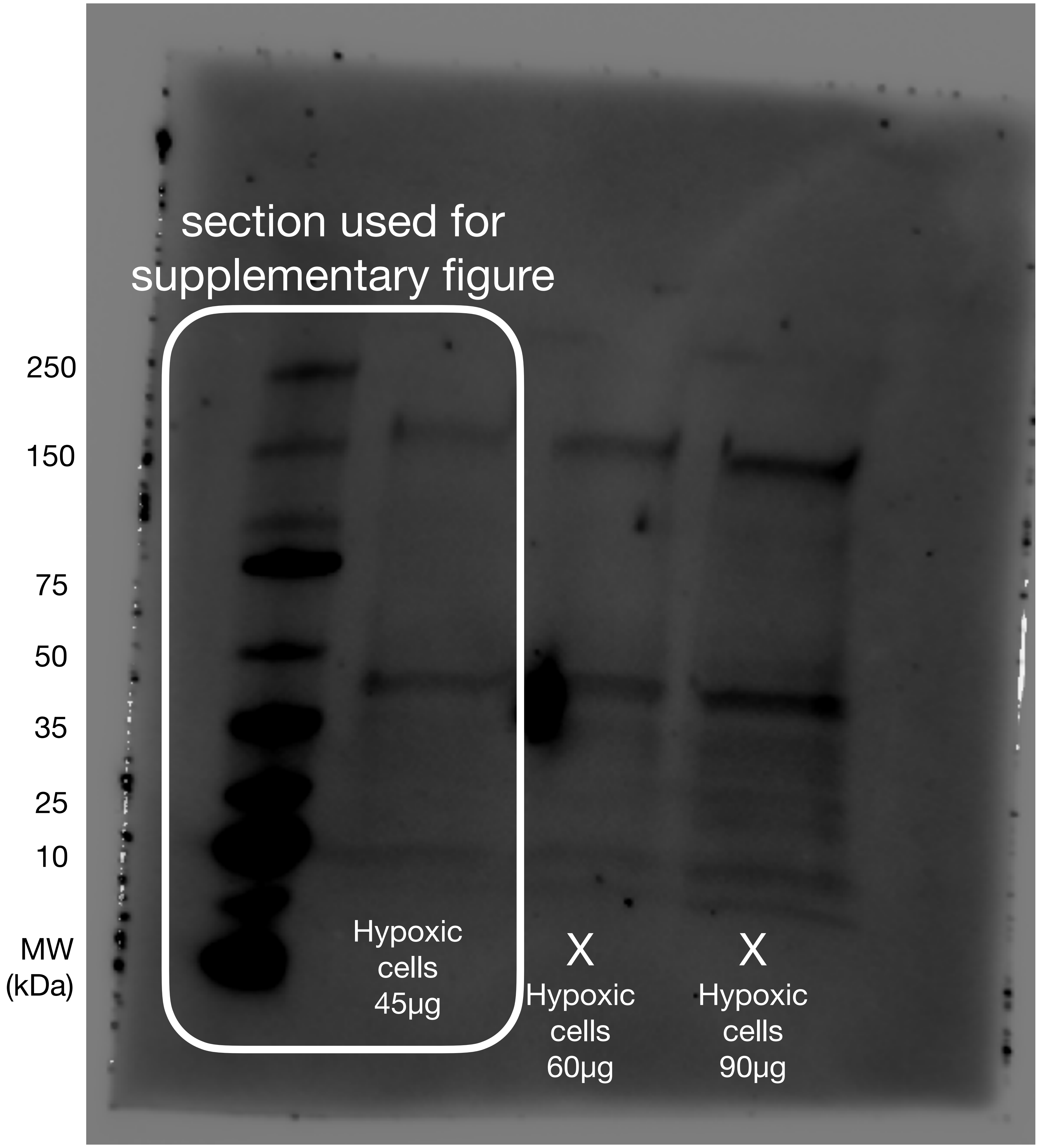

HIF monoclonal mouse- Mab1536 R&D systems
