# Supplementary figures and images for: The mouse gingiva and HIF-1α, a key gene of hypoxic environment, as tools for post-mortem time estimation
Source: PLoS One. 2024 Nov 15;19(11):e0311050. doi: 10.1371/journal.pone.0311050 (PMC11567532; doi:10.1371/journal.pone.0311050)

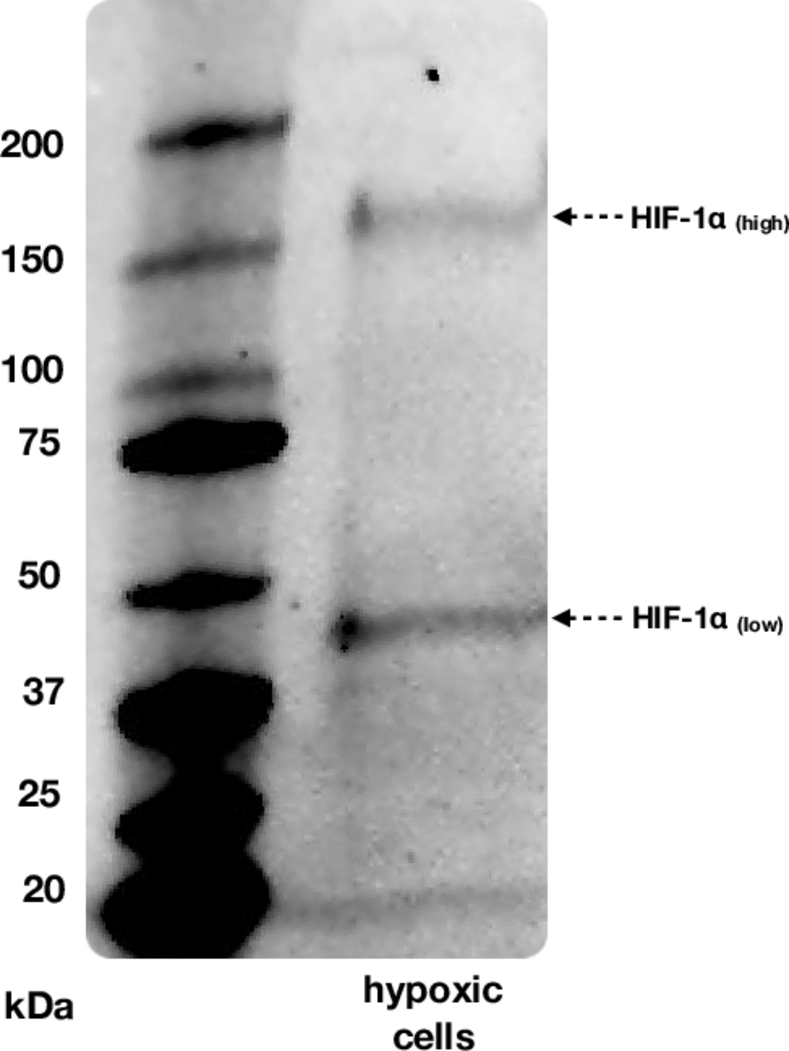

Supplement: S2 Fig — Western blot of HIF-1α protein expression: Membrane photograph of proteins from gingival cells isolated from living mice and cultivated under hypoxic conditions (5% O2), two bands corresponding to a high band of HIF-1α (150kDa) and low band of HIF-1α (50kDa). (TIF) [file pone.0311050.s002.tif]

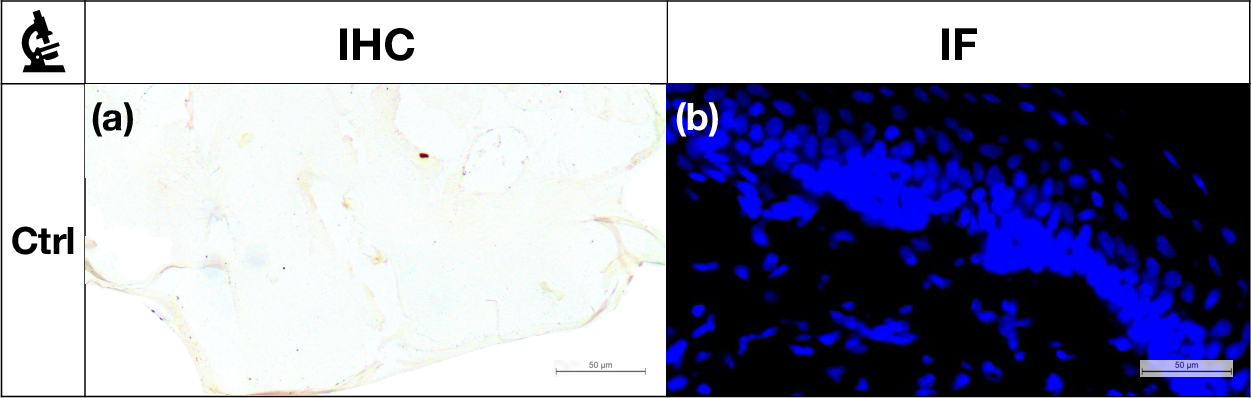

Supplement: S3 Fig — a) Negative control of immunohistochemistry with second antibody only and b) Negative control of immunofluorescence with second antibody only and DAPI labeling for nuclei. (TIF) [file pone.0311050.s003.tif]
